# Association between the use of midazolam, fentanyl, propofol, ketamine, and dexmedetomidine and the incidence of delirium in elderly patients in intensive care units: a systematic review

**DOI:** 10.1590/1516-3180.2024.0311R1.14072025

**Published:** 2025-10-06

**Authors:** Willian Setubal dos Santos, Omar Carrión-Torres, Matheus Galvão Valadares Bertolini Mussalem, Vinicius Santos Baptista, Samira Yarak

**Affiliations:** I Resident Physician, Universidade Federal de São Paulo (Unifesp), São Paulo (SP), Brazil.; II Resident Physician, Universidad Peruana de Ciencias Aplicadas (UPC), Santiago de Surco, Lima, Peru.; III Medical Student, Universidade Federal de S’o Paulo (Unifesp), S’o Paulo (SP), Brazil.; IV Medical Student, Universidade Federal de São Paulo (Unifesp), São Paulo (SP), Brazil.; V Professor, Universidade Federal de São Paulo (Unifesp), São Paulo (SP), Brazil.

**Keywords:** Delirium, Deep sedation, Intensive Care Units, Aged, Sedoanalgesics, ICU, Elderly, Dexmedetomidine, Propofol, Midazolam

## Abstract

**BACKGROUND:**

Delirium is a common and serious complication among elderly patients in intensive care units (ICUs), and is often associated with increased morbidity and mortality rates. The choice of sedoanalgesic may influence the incidence of delirium; however, the evidence remains unclear, particularly in the elderly population.

**OBJECTIVES:**

To evaluate the association between the use of different sedoanalgesics and the incidence of delirium in elderly ICU patients, based on data from randomized clinical trials.

**DESIGN AND SETTING:**

This systematic review was conducted using data from randomized clinical trials performed in various ICU settings.

**METHODS:**

A systematic search of the MEDLINE, Embase, and CENTRAL databases was performed in January 2024. The review included randomized clinical trials involving patients aged 60 years or older that examined the relationship between sedoanalgesics (midazolam, fentanyl, propofol, ketamine, and dexmedetomidine) and delirium incidence. Studies involving COVID-19 patients and non-randomized studies were excluded.

**RESULTS:**

A total of 1,331 patients from six studies were included. The mean age of the patients ranged from 71 to 74.7 years. Four studies compared dexmedetomidine with propofol; two found no significant difference in delirium incidence, whereas two suggested a lower incidence with dexmedetomidine. The remaining studies compared propofol with ketamine and dexmedetomidine with midazolam and showed no significant differences in the incidence of delirium.

**CONCLUSIONS:**

Dexmedetomidine may be associated with a lower incidence of delirium than propofol or midazolam in elderly ICU patients. However, further research is needed to confirm these findings and explore the factors contributing to delirium in this population.

**SYSTEMATIC REVIEW REGISTRATION:**

Registered with PROSPERO, CRD42024575693, available at https://www.crd.york.ac.uk/prospero/display_record.php?RecordID=575693.

## INTRODUCTION

 Delirium is a neuropsychiatric syndrome characterized by disturbances in cognition and consciousness, often observed in patients in intensive care units (ICUs), particularly among the elderly and those with prior cognitive impairment.^1,2^ The management of symptoms such as pain and agitation in ICUs typically involves sedation and anesthesia, which, while aimed at patient comfort, can contribute to the onset of delirium.^3,4^

 Midazolam, fentanyl, propofol, ketamine, and dexmedetomidine are among the most commonly used sedatives in Brazilian clinical practice, particularly in ICUs, due to their pharmacological properties suited for managing pain, agitation, and sedation in critically ill patients.^5,6^ The preference for these medications is influenced by their availability, cost-effectiveness, and specific characteristics, such as rapid onset of action and ease of titration.^7^ However, these agents are also associated with known adverse effects, including delirium, in certain clinical contexts.^8^

 The preference for these medications is influenced by their availability, cost-effectiveness, and specific characteristics, such as rapid onset of action and ease of titration.^7^ However, these agents are also associated with known adverse effects, including delirium, in certain clinical contexts.^8^

 This condition is exacerbated in elderly patients undergoing procedures requiring sedation, which increases the risk of cognitive impairment and consequently extends the duration and severity of delirium. These factors are associated with higher mortality.^9,10^ The presence of dementia or cognitive deficits in elderly individuals may further worsen patient orientation regarding their environment.^11^

 Delirium is associated with longer hospital stays, increased mortality, and long-term cognitive decline.^12^ Therefore, identifying sedatives and anesthetics that minimize the risk of delirium in this population is crucial for improving ICU care. The literature lacks a systematic review that specifically addresses this topic in the elderly population, despite it being the most affected by delirium. Therefore, this article reviewed randomized clinical trials to determine the relationship between various sedatives and the incidence of delirium in elderly ICU patients. 

## MATERIALS AND METHODS

### General information

 This study was a systematic review, and the manuscript was prepared in accordance with the Preferred Reporting Items for Systematic Reviews and Meta-Analyses (PRISMA) 2020 guidelines,^13^ with data searches conducted in January 2024. This study was published on the PROSPERO platform (ID No. CRD42024575693). The MEDLINE, Embase, and CENTRAL databases were used to identify studies on the incidence of delirium related to the use of sedoanalgesics in elderly ICU patients. The keywords employed were: (delirium) AND (aged) AND (intensive care unit) AND (propofol) OR (fentanyl) OR (dexmedetomidine) OR (ketamine) OR (midazolam), with adaptations according to the specificities of each database. 

### Data selection and extraction

 Article selection was conducted by two independent reviewers in two stages: an initial analysis of titles and abstracts, followed by full-text assessment. Discrepancies were resolved by consensus among all authors. The articles, unrestricted by language, were limited to publications published from 2000 onwards, to focus on recent data on sedoanalgesia in ICU settings. Only randomized clinical trials that examined patients aged 60 years or older using the Confusion Assessment Method for Intensive Care Units (CAM-ICU) to assess delirium were included. The choice of clinical trials is justified as they are the gold standard for investigating the relationship between delirium and the use of various sedoanalgesics, allowing for a rigorous analysis of the risks and safety of these drugs. The studied sedatives were midazolam, fentanyl, propofol, ketamine, and dexmedetomidine. 

 The exclusion criteria included articles that did not associate delirium with the use of specified sedoanalgesics, studies outside the ICU context, patients with COVID-19 or delirium tremens, cohorts, case reports, case-control studies, cross-sectional studies, reviews, and meta-analyses. These criteria aimed to eliminate potential confounding factors and ensure the relevance and accuracy of the analyzed data. It is important to note that the choice of inclusion and exclusion criteria preceded the search. 

 Data extraction was performed by two independent reviewers who collected information about the author, year, purpose, comparison, conclusion, type and location of the study, treatment interval, patient demographics, details of follow-up, intervention and control groups, drug dosing, incidence of delirium, odds ratio (OR), P-value, and sources of funding. 

### Methodological analysis

 In the methodology adopted in the present study, the Cochrane Risk of Bias Tool (RoB 2.0)^14^, which is a systematic approach for assessing bias in randomized clinical trials was used. The analysis focused on seven critical domains: the randomization process, allocation concealment, blinding of participants and personnel, blinding of outcome assessment, completeness of outcome data, addressing dropouts, selective reporting, and other potential sources of bias. 

 A thorough analysis was conducted on the adequacy of the random sequence generation process and allocation concealment to prevent predictability in participant selection. Lack of blinding and inadequate blinding of the outcome assessors were evaluated to mitigate the risk of performance and detection biases. Data integrity was rigorously verified, as was the selective reporting of outcomes. 

 The results of this bias assessment were communicated through an intuitive graph, in which the colored circles indicated the risk of bias in each domain per study, with green (low risk), yellow (uncertain risk), and red (high risk). This graph summarizes the risk profile of each study and aids in the critical interpretation of the results, ensuring a high standard of methodological rigor in the review. 

### Specifications

 In this study, effect measures such as odds ratio (OR) and relative risk (RR) were employed to assess the association between the use of analgesics and the incidence of delirium in elderly ICU patients. These measures were selected to facilitate quantitative comparison between the intervention and control groups across the included studies. OR was predominantly used to determine the likelihood of delirium occurrence following the administration of sedatives, such as dexmedetomidine, propofol, ketamine, and midazolam, thereby enabling a clear interpretation of the effects of these medications on the studied outcome. 

 For each synthesis, we tabulated the characteristics of the interventions and compared them with those of the planned groups. The data were prepared for synthesis by handling the missing summary statistics and converting them when necessary. The results of individual studies are displayed in structured tables and visual tools, such as risk-of-bias charts, to facilitate comparison. 

## RESULTS

### Study selection

**Figure 1:**
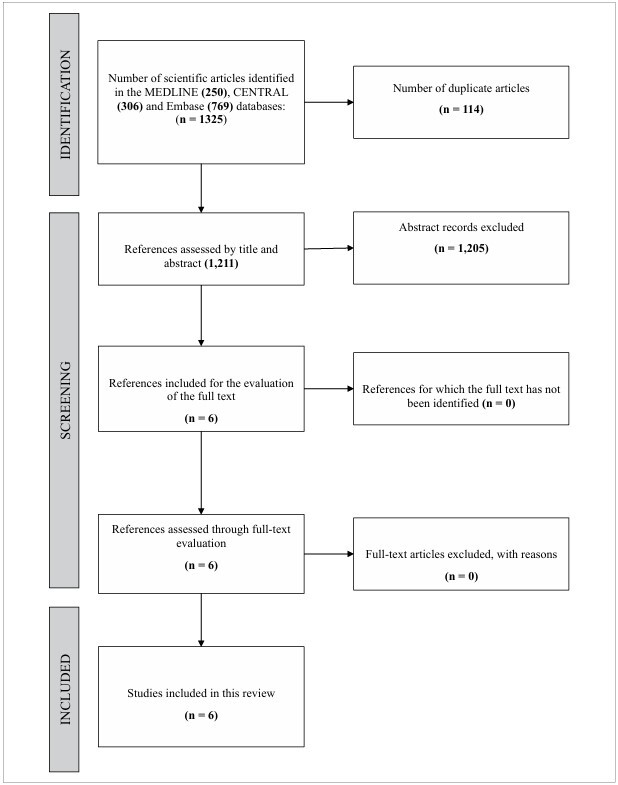
PRISMA flowchart.

 After implementing the search strategy across the databases, 1325 articles were identified: 250 from MEDLINE, 769 from Embase, and 306 from CENTRAL. Of these, 114 were duplicates and were subsequently removed. Following the analysis of the studies, 1,205 of the 1,211 articles were excluded for not meeting the inclusion criteria or meeting the exclusion criteria. The remaining six articles were read in full, and ultimately, all 6^15-20^ were selected for inclusion in this study. The reasons for excluding studies in the first stage of selection were drugs other than the five specified sedoanalgesics (which could introduce confounding bias in assessing the association between specific sedoanalgesic use and the occurrence of delirium), not using the CAM-ICU scale for delirium assessment, not associating the use of a specific sedoanalgesic with the occurrence of delirium, and not isolating the elderly population. These data are summarized in Figure 1. 

### Characteristics of the studies

 The six included studies were randomized clinical trials. The details of the objectives, comparisons, conclusions, and designs of the studies are presented in Table 1. Four studies^16,17,19,20^ compared dexmedetomidine with propofol. Another article^18^ compared dexmedetomidine and midazolam, whereas another study^15^ evaluated the use of ketamine versus propofol. No articles that compared the use of fentanyl with other sedoanalgesics or that met our inclusion criteria were found. Additionally, we did not find studies comparing dexmedetomidine with ketamine, propofol with midazolam, or ketamine with midazolam that met our inclusion criteria. 

**Table 1:** Characteristics and summary of the studies

**Reference**	**Objective**	**Comparison**	**Conclusion**	**Study design**
Siripoonyothai S et al.^15^ Thailand	To compare postoperative delirium between ketamine and propofol during cardiac surgery with the use of extracorporeal circulation machine.	Postoperative delirium between groups that received ketamine and propofol.	Compared to propofol, ketamine resulted in fewer delirium events. However, the results are inconclusive.	Randomized clinical trial
Djaiani G et al.^16^ Canada	Comparing dexmedetomidine and propofol regarding the incidence of postoperative delirium in elderly patients after cardiac surgery.	Postoperative delirium between sedation with dexmedetomidine and propofol.	Sedation with dexmedetomidine reduced the incidence, delayed the onset, and shortened the duration of postoperative delirium compared to propofol.	Randomized clinical trial
Shi C et al.^17^ China	Investigating the effect of perioperative administration of dexmedetomidine on the occurrence and duration of postoperative delirium in elderly patients after cardiac surgery.	Postoperative occurrence of delirium between sedation with dexmedetomidine and propofol.	There was no significant difference in the incidence of delirium between the dexmedetomidine and propofol groups.	Randomized clinical trial
Shu A et al.^18^ China	Investigating the influence of dexmedetomidine on delirium and hemodynamics in elderly patients under mechanical ventilation in the ICU.	Anti-delirium effects and hemodynamic influence between dexmedetomidine and midazolam.	Compared to midazolam, dexmedetomidine showed a lower incidence of delirium. However, there was no significant difference between the groups.	Randomized clinical trial
Fang H et al.^19^ China	Assessing the sedative effect and safety of dexmedetomidine for postoperative mechanical ventilation in elderly patients.	Sedation effects and safety of dexmedetomidine compared to propofol.	It does not reduce the incidence of delirium or ICU length of stay.	Randomized clinical trial
Shin HJ et al.^20^ South Korea	Evaluating the incidence of postoperative delirium in elderly patients undergoing lower limb orthopedic surgery under spinal anesthesia.	Postoperative occurrence of delirium between sedation with dexmedetomidine and propofol.	Dexmedetomidine showed a lower incidence of delirium than propofol.	Randomized clinical trial

Description: ICU, intensive care unit.

 It is also noteworthy that among the six included studies, four received funding for their research, whereas the other two did not report any funding in their manuscripts.^15-20^

### Demographic analysis

 A total of 1,331 patients were evaluated in the six studies included in this review, comprising 561 men and 770 women. Additionally, three studies assessed patients over 60 years old, two studies examined patients over 65 years old, and one study focused on patients between 65 and 80 years of age.^15-20^ The average age of the patients ranged from 71 to 74.7 years, although one of the studies did not report the average age of the participants.^15^ The overall average age of the articles evaluated, considering the five studies that reported this value, was 72.37 years. No study has reported data on the median age of the patients. The selected studies were published between 2014 and 2023 and patient evaluations were conducted between 2009 and 2021. These studies were conducted in Thailand, China, Canada, and South Korea. 

 The average follow-up time was reported in three studies, with two of them noting a duration of five days, while the other reported a period of three days.^16,17,20^ The follow-up time was limited to the period in which these patients were hospitalized in an ICU, and specifics on how delirium assessments were conducted during this follow-up are shown in Table 2. 

**Table 2:** Patient characteristics

**Reference**	**Treatament (year)**	**Patients (n)**	**Men (n)**	**Women (n)**	**Population (year)**	**Mean Age (year ± SD)**	**Follow-up (day)**	**Follow-up details**
Siripoonyothai S et al.^15^	2019–2020	64	35	29	> 65	NR	NR	Assessment of postoperative delirium within the first 24 hours in the ICU
Djaiani G et al.^16^	2011–2014	183	138	45	> 60	Intervention: 72,7 (6,4) Control: 72,4 (6,2)	5	Delirium assessment was conducted every 12 hours or as needed, using the CAM-ICU tool
Shi C et al.^17^	2009–2016	164	119	45	> 60	Intervention: 74,7 (7,2) Control: 74,2 (7,7)	5	Delirium assessment was performed twice daily until the fifth day post-surgery, utilizing the CAM-ICU assessment method
Shu A et al.^18^	2015–2016	80	52	28	> 60	Intervention: 73,4 (8,6) Control: 73,8 (7,9)	NR	Delirium assessment was conducted during the ICU stay
Fang H et al.^19^	2011–2012	108	62	46	65–80	Intervention: 73,7 (3,1) Control: 74,2 (4,2)	NR	Observation during mechanical ventilation and ICU admission
Shin HJ et al.^20^	2017–2021	732	155	577	> 65	Intervention: 71 (NR) Control: 72 (NR)	3	Delirium assessment using the confusion assessment method in the first three postoperative days

Description: NR, not reported; SD, standard deviation; CAM-ICU, Confusion Assessment Method for Intensive Care Units; ICU, intensive care unit.

### Methodological analysis and risk of bias

**Figure 2:**
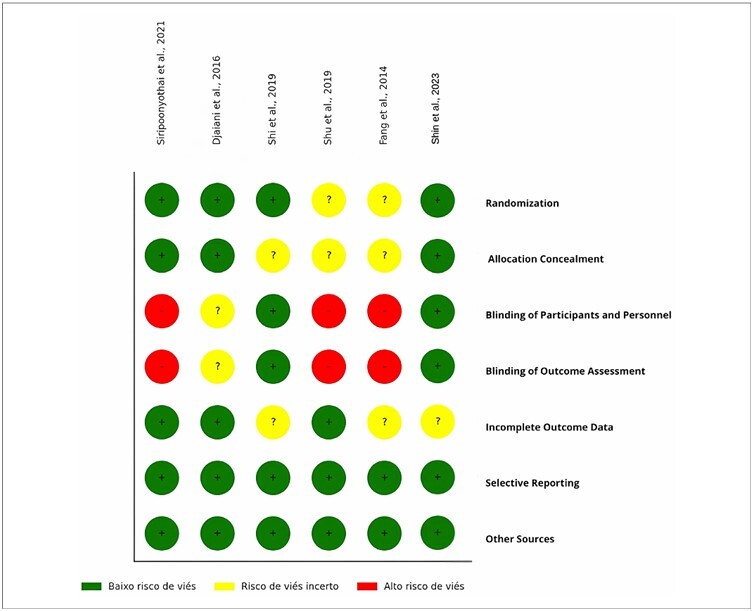
Bias risk analysis.

 The methodological analysis of the included studies indicated that most adhered to rigorous procedures for randomization and allocation concealment, as demonstrated by Siripoonyothai and Sindhvananda^15^ and Shin et al.^20^ However, there was a lack of clarity in the blinding of participants and assessors in some studies, including those by Siripoonyothai and Sindhvananda^15^ and Shu et al.,^18^ introducing uncertainty in this aspect (Figure 2). 

 Djaiani et al.^16^ effectively managed allocation concealment, despite employing single blinding. Fang et al.^19^ also presented deficiencies in detailing the blinding procedures, resulting in an uncertain risk. The analysis of randomized patients was consistently well-conducted, indicating appropriate handling of incomplete data. Shu et al.^18^ demonstrated a low risk of bias in the blinding. 

 The study by Shin et al.^20^ with its double-blind design and rigorous postoperative data collection procedures, effectively minimized performance and detection biases, although the absence of a pre-registered protocol leaves the risk of selective reporting uncertain. Figure 2 highlights the predominance of a low bias risk across several critical categories, emphasizing the need for careful evaluation of the blinding of participants, personnel, and outcome assessors. 

### Sedoanalgesics and delirium

 Four studies compared dexmedetomidine with propofol.^16,17,19,20^ Two of these studies found no statistically significant differences in delirium rates between the groups, with P values of 0.0758 and 0.434 (95% confidence interval [95% CI]).^17,19^ Shi et al.^17^ compared 84 patients receiving dexmedetomidine (0.4–0.6 μg/[kg h]) with 80 patients receiving propofol (25–50 mg/[kg h]), reporting delirium incidences of 39.3% and 26.3%, respectively. Fang et al.^19^ compared 54 patients on dexmedetomidine (0.20.7 μg/[kg h]) with 54 patients on propofol (0.3–4 mg/[kg h]), with delirium incidences of 3.7% and 9.2%, respectively. 

 In contrast, the other two studies found significant differences in delirium incidence.^16,20^ Djaiani et al.^16^ compared 91 patients on dexmedetomidine (0.4 μg/kg bolus, followed by 0.2–0.7 μg/[kg h]) with 92 patients on propofol (1.5–3 mg/[kg h]), reporting delirium incidences of 17.5% and 31.5%, respectively (P = 0.028, 95% CI, OR = 0.46, interval 0.23–0.92). Shin et al.^20^ compared 366 patients on dexmedetomidine (1 μg/kg bolus, followed by 0.10.5 μg/[kg h]) with 366 patients on propofol (1–2 μg/ml infusion), with incidences of 3% and 6%, respectively (P = 0.036, 95% CI, OR = 0.42, interval 0.20–0.86). 

 Siripoonyothai and Sindhvananda^15^ compared propofol with ketamine and found no significant difference in the incidence of delirium between the groups. The study involved 32 patients on propofol (1.5–6 mg/[kg h]) and 32 on ketamine (1 mg/[kg·h]), with delirium incidences of 56.25% and 31.25%, respectively (P = 0.47, 95% CI, OR = 2.11, interval 0.29–26.42). 

 Shu et al.^18^ compared dexmedetomidine and midazolam and found no significant difference in the incidence of delirium. The study compared 40 patients on dexmedetomidine (1 μg/kg bolus, followed by 0.2–0.7 μg/[kg h]) with 40 patients on midazolam (0.05 mg/kg bolus, followed by 0.05–0.10 mg/[kg h]), reporting delirium incidences of 0% and 10%, respectively (P = 0.116, 95% CI). The odds ratios for this outcome have not been reported. The relevant data are summarized in Tables 3 and 4. 

**Table 3:** Sedoanalgesia details

**Reference**	**Intervention group**	**Control group**	**Intervention (n)**	**Control (n)**	**Dose – Intervention**	**Dose – Control**
Siripoonyothai S et al.^15^	Propofol	Ketamine	32	32	Infusion of propofol ranging from 1.5–6 mg/(kg h)	Infusion of ketamine at 1 mg/(kg h)
Djaiani G et al.^16^	Dexmedetomidine	Propofol	91	92	Bolus of 0.4 μg/kg of dexmedetomidine followed by an infusion of 0.2–0.7 μg/(kg h).	Infusion of propofol ranging from 1.5–3 mg/(kg h)
Shi C et al.^17^	Dexmedetomidine	Propofol	84	80	Infusion of dexmedetomidine ranging from 0.4–0.6 μg/(kg h)	Infusion of propofol ranging from 25–50 mg/(kg h)
Shu A et al.^18^	Dexmedetomidine	Midazolam	40	40	Bolus of 1 μg/kg of dexmedetomidine followed by an infusion of 0.2–0.7 μg/(kg h)	Bolus of 0.05 mg/kg of midazolam followed by an infusion of 0.05–0.10 mg/(kg h)
Fang H et al.^19^	Dexmedetomidine	Propofol	54	54	Infusion of dexmedetomidine ranging from 0.2–0.7 μg/(kg h)	Infusion of propofol ranging from 0.3–4 mg/(kg h)
Shin HJ et al.^20^	Dexmedetomidine	Propofol	366	366	Bolus of 1 μg/kg of dexmedetomidine followed by an infusion of 0.1–0.5 μg/(kg h)	Continuous infusion of propofol adjusted to an effective concentration of 1–2 μg/ml

**Table 4:** Analysis of the incidence of delirium

**Reference**	**Presence of delirium Intervention (n)**	**Presence of delirium Control (n)**	**Odds Ratio**	**P value**
Siripoonyothai S et al.^15^	18 of 32 patients (56,25%)	10 of 32 patients (31,25%)	2,11 (0,29–26,42)	0,47
Djaiani G et al.^16^	16 of 91 patients (17,5%)	29 of 92 patients (31,5%)	0,46 (0,23–0,92)	0,028
Shi C et al.^17^	33 of 84 patients (39,3%)	21 of 80 patients (26,3%)	NR	0,0758
Shu A et al.^18^	0 of 40 patients (0%)	4 of 40 patients (10%)	NR	0,116
Fang H et al.^19^	2 of 54 patients (3,7%)	5 of 54 patients (9,2%)	NR	0,434
Shin HJ et al.^20^	11 of 366 patients (3%)	24 of 366 patients (6%)	0,42 (0,20–0,86)	0,036

Description: NR, not reported.

## DISCUSSION

### General considerations

 The association between sedoanalgesia and the onset of delirium in ICU patients, particularly the elderly, is well documented; however, its multifactorial etiology complicates targeted intervention strategies.^21-25^ Delirium incidence significantly increases with age, particularly post-65 years, reflecting the heightened vulnerability of this demographic to ICU-related stressors and pre-existing neuropsychiatric conditions.^26-29^ Given this, our review focused exclusively on studies involving elderly patients to address the nuances of this high-risk group. 

 The choice of the diagnostic tool critically influences the consistency and reliability of delirium detection. The use of the CAM-ICU scale across all included studies ensured a standardized approach, although variations in assessment intervals were noted, ranging from twelve to twenty-four hours, or unspecified in some cases.^30-35^ These discrepancies underline the need for uniform application protocols to enhance the comparability of study outcomes. 

### Dexmedetomidine versus propofol

 In the comparative analysis between dexmedetomidine and propofol, our findings suggest that dexmedetomidine may provide a protective effect against delirium, which is supported by the significant results of two of the four studies reviewed.^16,20^ Specifically, Djaiani et al.^16^ and Shin et al.^20^ reported a reduced incidence of delirium with dexmedetomidine, indicating its potential advantage in certain clinical scenarios, such as orthopedic surgery. The high methodological quality of these studies, as reflected in their RoB ratings, enhances the credibility of their results. 

 However, this protective effect was not observed uniformly across all studies. Shi et al.^17^ and Fang et al.^19^ found no significant differences in the incidence of delirium between dexmedetomidine and propofol, suggesting that variations in dosage and bolus administration may critically influence outcomes. 

 Although this review did not include placebo-controlled trials, existing evidence emphasizes the importance of administration techniques in maximizing the protective effects of dexmedetomidine and improving long-term cognitive outcomes.^36-40^ Consequently, future research should focus on precise dosing and standardized administration to better define the role of dexmedetomidine in delirium prevention and provide clearer guidance for sedation practices in ICU settings, ultimately aiming to optimize patient outcomes. 

### Dexmedetomidine versus midazolam

 Shu et al.^18^ observed that although there was no statistically significant difference between the groups, the dexmedetomidine group exhibited a descriptively lower incidence of delirium than the midazolam group. This lack of statistical significance may be attributed to the small sample size studied.^41-47^ In contrast, the literature comparing these two drugs in both adult and elderly populations (without isolating the elderly) consistently shows that dexmedetomidine has a protective effect against delirium, while midazolam is associated with a higher incidence of this outcome. 

 Further supporting this, Pasin et al.^43^ systematic review and meta-analysis of 14 randomized clinical trials involving 3,029 adult patients found that dexmedetomidine significantly reduced the occurrence of delirium, agitation, and confusion compared to the control groups (P = 0.03, 95% CI, RR = 0.68, interval 0.49–0.96). Although this meta-analysis did not exclusively focus on elderly patients, the evidence strongly suggests that dexmedetomidine may be beneficial in reducing these adverse outcomes. 

 Given the findings of Shu et al.^18^ and the broader literature, it is evident that larger and more targeted studies specifically focusing on the elderly population are necessary. These studies should have sufficient sample sizes to reduce random errors and enhance the reliability of the conclusions.^48^ Such research is crucial to fill the current gap in the literature and provide more precise guidance on the use of sedatives, such as dexmedetomidine, in elderly patients, ultimately leading to improved clinical outcomes in this vulnerable group. 

### Propofol versus ketamine

 The comparison between propofol and ketamine, as explored by Siripoonyothai and Sindhvananda,^15^ did not yield conclusive results owing to wide confidence intervals and potential sample size limitations. The observed trends suggest a higher incidence of delirium with propofol; however, the variability in the data underscores the need for more robust trials to definitively establish the comparative risks of these sedatives. Moreover, there is a lack of consensus on the impact of ketamine on delirium in both adult and elderly populations, as evidenced by Shurtleff et al.,^49^ further emphasizing the need for comprehensive research to clarify its effects across different age groups. 

### Final considerations

 The high heterogeneity across the included studies due to differences in population demographics, sedative regimens, and outcome measurements precluded a meta-analysis. However, despite these variations, the pooled data provided valuable insights into the relationship between sedoanalgesics and delirium. Notably, significant gaps in the literature were identified, including the absence of studies comparing fentanyl with other sedatives and the lack of research on drug combinations, such as propofol and midazolam, propofol and ketamine, or midazolam and ketamine. These deficiencies, particularly in older adults, underscore the urgent need for comprehensive studies to evaluate these factors. Such research is essential for developing more effective and tailored sedation strategies in ICU settings and for providing conclusive and detailed evidence regarding the comparative efficacy and safety profiles of these drugs. 

### Strengths and limitations

 This review aimed to synthesize data from clinical trials available in the literature since 2000 on the incidence of delirium and its relationship with the use of sedoanalgesics in elderly ICU patients. The methodology used is detailed to allow for replication in this study. This review maintains a high scientific standard by adhering to all the Cochrane recommendations. The extraction of descriptive and statistical data enabled a broader assessment of the included studies. 

 The methodology, restricted to clinical trials focusing on the elderly, has limited the availability of relevant literature for analysis. To compensate for this limitation, literature on the impact of these drugs on the general adult population is included in the discussion. Although this may result in less specific conclusions for the elderly, the combined approach underscores the need for more research dedicated to this age group to avoid inappropriate generalizations between studies of different populations. 

 Additionally, the deliberate exclusion of studies comparing drug use with placebo aimed to maintain a focus on the comparative analysis of different pharmacological therapies employed in hospital settings. This decision seeks to better understand the current practices and therapeutic options available for sedoanalgesia among elderly populations. 

 This systematic review did not identify randomized clinical trials conducted in Brazil that met the inclusion criteria. Consequently, we also included studies conducted in other countries, such as Thailand, China, Canada, and South Korea, that explored strategies for sedoanalgesia in elderly patients in ICU settings and employed the same pharmacological agents widely used in Brazilian intensive care practice. This approach enabled the synthesis of relevant evidence for clinical practice despite contextual differences. Nevertheless, we highlight the need for future studies to evaluate sedoanalgesia protocols within the Brazilian context, considering local specificities, and complementing the international experience presented in this review regarding delirium outcomes in elderly patients. 

## CONCLUSION

 This study highlights the complex relationship between sedative use and the occurrence of delirium in elderly ICU patients, emphasizing that advanced age is a significant risk factor for delirium. Despite the varying results, dexmedetomidine appears to be potentially more beneficial than propofol and midazolam. The limitations of the studies in the literature underscore the need for more comprehensive and standardized future research. The variability in sedative dosages and administration methods further highlights the importance of additional clinical trials to establish optimized sedation practices that minimize the risk of delirium, thereby improving care and outcomes in this vulnerable population. 

## References

[1] American Psychiatric Association (2022). Diagnostic and statistical manual of mental disorders (DSM-5-TR) [Internet].

[2] Vasilevskis EE, Han JH, Hughes CG, Ely EW (2012). Epidemiology and risk factors for delirium across hospital settings. Best Pract Res Clin Anaesthesiol.

[3] Pun BT, Balas MC, Barnes-Daly MA (2019). Caring for critically ill patients with the ABCDEF bundle: results of the ICU liberation collaborative in over 15,000 adults. Crit Care Med.

[4] Pereira JV, Sanjanwala RM, Mohammed MK, Le ML, Arora RC (2020). Dexmedetomidine versus propofol sedation in reducing delirium among older adults in the ICU: a systematic review and meta-analysis. Eur J Anaesthesiol.

[5] Carmo TG (2017). Vantagens e desvantagens do uso de dexmedetomidina na sedação em unidades de terapia intensiva. Revista Saúde e Desenvolvimento.

[6] Sakata RK (2010). Analgesia e sedação em unidade de terapia intensiva. Revista Brasileira de Anestesiologia [Internet].

[7] Skrobik Y (2015). Chasing the elusive notion of delirium causality. Intensive Care Med.

[8] Muellejans B, Matthey T, Scholpp J, Schill M (2006). Sedation in the intensive care unit with remifentanil/propofol versus midazolam/fentanyl: a randomised, open-label, pharmacoeconomic trial. Crit Care.

[9] Bisinotto FMB, Silveira LAM, Silva RO, Martins LB (2017). Postoperative delirium in the elderly: where are we?. Revista Médica de Minas Gerais.

[10] Khan BA, Perkins AJ, Prasad NK (2020). Biomarkers of delirium duration and delirium severity in the ICU. Crit Care Med.

[11] Herling SF, Greve IE, Vasilevskis EE (2018). Interventions for preventing intensive care unit delirium in adults.. Cochrane Database Syst Rev.

[12] Campbell NL, Perkins AJ, Khan BA (2019). Deprescribing in the pharmacologic management of delirium: a randomized trial in the intensive care unit. Journal of the American Geriatrics Society.

[13] Page MJ, McKenzie JE, Bossuyt PM (2021). The PRISMA 2020 statement: an updated guideline for reporting systematic reviews. Syst Rev.

[14] Higgins JP, Savović J, Page MJ, Elbers RG, Sterne JA (2019). Chapter 8: Assessing risk of bias in a randomized trial. Cochrane [Internet].

[15] Siripoonyothai S, Sindhvananda W (2021). Comparison of postoperative delirium within 24 hours between ketamine and propofol infusion during cardiopulmonary bypass machine: a randomized controlled trial. Ann Card Anaesth.

[16] Djaiani G, Silverton N, Fedorko L (2016). Dexmedetomidine versus propofol sedation reduces delirium after cardiac surgery: a randomized controlled trial. Anesthesiology.

[17] Shi C, Jin J, Qiao L (2019). Effect of perioperative administration of dexmedetomidine on delirium after cardiac surgery in elderly patients: a double-blinded, multi-center, randomized study. Clin Interv Aging.

[18] Shu A, Fu Y, Luo Y (2019). An investigation on delirium and hemodynamics influenced by dexmedetomidine for sedating elderly patients in mechanical ventilation. Int J Clin Exp Med [Internet].

[19] Fang H, Jun W, Xinjing Y (2014). Analysis of the sedetative effect of dexmedetomidine on postoperative mechanical ventilation in elderly patients. Chinese Medical Journal.

[20] Shin HJ, Woo Nam S, Kim H (2023). Postoperative delirium after dexmedetomidine versus propofol sedation in healthy older adults undergoing orthopedic lower limb surgery with spinal anesthesia: a randomized controlled trial. Anesthesiology.

[21] Pisani MA, Murphy TE, Araujo KL (2009). Benzodiazepine and opioid use and the duration of intensive care unit delirium in an older population. Crit Care Med.

[22] Almeida TM, Azevedo LC, Nosé PM, Freitas FG, Machado FR (2016). Risk factors for agitation in critically ill patients. Rev Bras Ter Intensiva.

[23] Jaber S, Chanques G, Altairac C (2005). A prospective study of agitation in a medical-surgical ICU: incidence, risk factors, and outcomes. Chest.

[24] Fraser GL, Prato BS, Riker RR, Berthiaume D, Wilkins ML (2000). Frequency, severity, and treatment of agitation in young versus elderly patients in the ICU. Pharmacotherapy.

[25] Prayce R, Quaresma F, Galriça I (2018). Delirium: o 7º parâmetro vital? [Delirium: the 7th vital sign?]. Acta Med Port.

[26] Pandharipande P, Shintani A, Peterson J (2006). Lorazepam is an independent risk factor for transitioning to delirium in intensive care unit patients. Anesthesiology.

[27] Tilouche N, Hassen MF, Ali HBS (2018). Delirium in the intensive care unit: incidence, risk factors, and impact on outcome. Indian J Crit Care Med.

[28] Pisani MA, Murphy TE, Araujo KL (2009). Benzodiazepine and opioid use and the duration of intensive care unit delirium in an older population. Crit Care Med.

[29] Li X, Zhang L, Gong F, Ai Y (2020). Incidence and risk factors for delirium in older patients following intensive care unit admission: a prospective observational study. J Nurs Res.

[30] Micek ST, Anand NJ, Laible BR, Shannon WD, Kollef MH (2005). Delirium as detected by the CAM-ICU predicts restraint use among mechanically ventilated medical patients. Crit Care Med.

[31] Roberts B, Rickard CM, Rajbhandari D (2005). Multicentre study of delirium in ICU patients using a simple screening tool. Aust Crit Care.

[32] Ely EW, Margolin R, Francis J (2001). Evaluation of delirium in critically ill patients: validation of the Confusion Assessment Method for the Intensive Care Unit (CAM-ICU). Crit Care Med.

[33] Gusmao-Flores D, Salluh JI, Chalhub RÁ, Quarantini LC (2012). The Confusion Assessment Method for the Intensive Care Unit (CAM-ICU) and intensive care delirium screening checklist (ICDSC) for the diagnosis of delirium: a systematic review and meta-analysis of clinical studies. Crit Care.

[34] Guenther U, Popp J, Koecher L (2010). Validity and reliability of the CAM-ICU flowsheet to diagnose delirium in surgical ICU patients. J Crit Care.

[35] Soja SL, Pandharipande PP, Fleming SB (2008). Implementation, reliability testing, and compliance monitoring of the Confusion Assessment Method for the Intensive Care Unit in trauma patients. Intensive Care Medicine.

[36] Zhang DF, Su X, Meng ZT (2019). Impact of dexmedetomidine on longterm outcomes after noncardiac surgery in elderly: 3-year followup of a randomized controlled trial. Ann Surg.

[37] Deiner S, Luo X, Lin HM (2017). Intraoperative infusion of dexmedetomidine for prevention of postoperative delirium and cognitive dysfunction in elderly patients undergoing major elective noncardiac surgery: a randomized clinical trial. JAMA Surg.

[38] Li CJ, Wang BJ, Mu DL (2020). Randomized clinical trial of intraoperative dexmedetomidine to prevent delirium in the elderly undergoing major non-cardiac surgery. Br J Surg.

[39] Guo Y, Sun LL, Chen ZF, Li QF, Jiang H (2015). [Preventive effect of dexmedetomidine on postoperative delirium in elderly patients with oral cancer]. Shanghai Kou Qiang Yi Xue.

[40] Priye S, Jagannath S, Singh D, Shivaprakash S, Reddy DP (2015). Dexmedetomidine as an adjunct in postoperative analgesia following cardiac surgery: a randomized, double-blind study. Saudi J Anaesth.

[41] Maldonado JR, Wysong A, van der Starre PJ (2009). Dexmedetomidine and the reduction of postoperative delirium after cardiac surgery. Psychosomatics.

[42] Keating GM (2015). exmedetomidine: a review of its use for sedation in the intensive care setting. Drugs.

[43] Pasin L, Landoni G, Nardelli P (2014). Dexmedetomidine reduces the risk of delirium, agitation and confusion in critically Ill patients: a meta-analysis of randomized controlled trials. J Cardiothorac Vasc Anesth.

[44] Wen J, Ding X, Liu C (2023). A comparation of dexmedetomidine and midazolam for sedation in patients with mechanical ventilation in ICU: a systematic review and meta-analysis. PLoS One.

[45] Riker RR, Shehabi Y, Bokesch PM, SEDCOM (Safety and Efficacy of Dexmedetomidine Compared With Midazolam) Study Group (2009). Dexmedetomidine vs midazolam for sedation of critically ill patients: a randomized trial. JAMA.

[46] Wan LJ, Huang QQ, Yue JX, Lin L, Li SH (2011). [Comparison of sedative effect of dexmedetomidine and midazolam for post-operative patients undergoing mechanical ventilation in surgical intensive care unit]. Zhongguo Wei Zhong Bing Ji Jiu Yi Xue.

[47] Sampurnanand, Chilana D, Sinha AK (2023). A comparative study of dexmedetomidine and midazolam for sedation in patients on mechanical ventilation in ICU. Int J Acad Med Pharm (JAMP).

[48] Schulz KF, Grimes DA (2005). Sample size calculations in randomised trials: mandatory and mystical. Lancet.

[49] Shurtleff V, Radosevich JJ, Patanwala AE (2020). Comparison of ketamine- versus nonketamine-based sedation on delirium and coma in the intensive care unit. J Intensive Care Med.

